# OD02 Developing Components For A National Strategy For Heart Valve Disease In Canada

**DOI:** 10.1017/S0266462324001405

**Published:** 2025-01-07

**Authors:** John M Sproule, Lindsey Warkentin, David Messika-Zeitoun

## Abstract

**Introduction:**

The research included a rapid review of current literature to describe epidemiology, management, and system impact of heart valve disease (HVD) in adult populations. Key issues were identified in consultation with expert focus groups and were framed across the continuum of care and systemic policy issues. The groupings were adapted and adjusted during deliberations.

**Methods:**

A rapid literature review was conducted on HVD key interventions and evidence of effectiveness along with expert interviews to identify high-level themes for reform. This served as the evidentiary backgrounder. Two virtual policy engagements with clinical leaders, patients, and health system managers were conducted. The focus was their collective drafting of recommendations. These workshops identified nine thematic areas and developed associated recommendations for action under each theme. The success of the process is evident as the report has been taken up as a roadmap for ongoing research and policy work.

**Results:**

A comprehensive grouping of recommendations for improving HVD detection, management, and treatment in Canada was produced. It was designed to be comprehensive to then allow more targeted work to proceed under an evidence-informed and clinically endorsed agenda.

**Conclusions:**

Heart valve conditions are increasingly treatable, especially if detected early. Innovation in treatments as well as detection and management to address gaps in care were identified as the most urgent priorities. The key result was formation of formal working groups with a professional society to explore spoke–hub-and node models for care delivery and to launch awareness programs for early detection.

